# In silico tools for mechanical analysis of extra‐ and intra‐luminal artificial urinary sphincters

**DOI:** 10.1002/bco2.473

**Published:** 2024-12-25

**Authors:** Gianluca Mazzucco, Paola Pirini, Chiara Giulia Fontanella, Alice Berardo, Maria Vittoria Mascolini, Ilaria Toniolo, Leonardo Marziale, Tommaso Mazzocchi, Gioia Lucarini, Nicolò Spiezia, Emanuele Luigi Carniel

**Affiliations:** ^1^ Centre for Mechanics of Biological Materials University of Padova Padova Italy; ^2^ Department of Civil, Environmental and Architectural Engineering University of Padova Padova Italy; ^3^ Department of Industrial Engineering University of Padova Padova Italy; ^4^ Relief Srl Pontedera Italy; ^5^ The BioRobotics Institute Scuola Superiore Sant'Anna Pontedera Italy; ^6^ M3E Srl Padova Italy

**Keywords:** artificial urinary sphincter, computational biomechanics, in silico medicine, mechanical stimulation of urethral tissues, urinary incontinence

## Abstract

**Objectives:**

To analyse and compare the functionality of extraluminal and intraluminal artificial urinary sphincters (AUSs), an in silico procedure has been defined and applied. Design and reliability assessments of the AUS are typically performed using a clinical approach, which does not provide data on mechanical stimulation of urethral tissues. Mechanical stimulation may determine tissue degeneration, such as urethral atrophy or erosion, the main causes of AUS failure. In silico techniques can provide a quantitative description of stress and strain fields due to the interaction between tissues and AUS and allow investigating an extremely large number of situations, considering different configurations of AUS and urethra.

**Materials and Methods:**

Computational investigations were carried out to evaluate the mechanical reliability of the main extraluminal and intraluminal AUS, AMS 800 and Relief. The lower urinary tract was modelled based on previous experiments. The AUS models took into account the main components that interact with biological tissues. Urethra and AUS models were coupled and used to investigate mechanical stimulation of urethral tissues.

**Results:**

In silico simulations provide quantitative information about the mechanical stimulation of urethral tissue, such as compressive strain and stress and hydrostatic pressure, due to interaction with the AUS. Such mechanical quantities allow a comparison of reliability between extraluminal and intraluminal devices.

**Conclusions:**

The activities define and demonstrate the effectiveness of a novel in silico approach to the design and reliability assessment of AUS devices, increasing the investigative possibilities and reducing the time, ethical and economic costs.

## 1 INTRODUCTION

The huge diffusion of urinary incontinence (UI) entails enormous social and economic costs.^1^, ^2^, ^3^ Despite artificial urinary sphincter (AUS) is the gold standard treatment for severe UI, intervention failure frequently occurs because of tissue vaso‐constriction, infection, atrophy and/or erosion.^4^, ^5^, ^6^, ^7^ Excessive mechanical stimulation of urethral tissues entails such phenomena, but usually it is not investigated.^8^ Indeed, the traditional AUS design and reliability assessment is based on the classical clinical approach, such as experiments on human cadavers and animal models, and clinical trials.

On the other hand, the in silico approach to the design and reliability assessment of surgical and prosthetic devices is beginning to take hold.^9^, ^10^, ^11^, ^12^ Such bioengineering methods, which involve coupled experimental and computational activities, require the development of mathematical models of the system, such as the biological structure and the prosthetic device. Experimental activities are essential for the definition, development and identification of models. Subsequently, computational methods allow the extension of experimental results to a much broader scenario by considering different configurations of the system and boundary conditions, such as different conformations of the anatomical site, different configurations of the prosthetic device and multiple surgical procedures. In addition, computational biomechanics provides data that are difficult to obtain from experimental and clinical activities, such as stress and strain fields within biological tissues. Such mechanical stimuli determine a variety of mechanobiological phenomena such as vasoconstriction, tissue damage, remodelling and signal mechano‐transduction.^13^, ^14^

Specifically for AUS devices, in silico models can evaluate a large number of device configurations as well as anatomical and histological conformations of the lower urinary tract, especially in pathological situations.^8^, ^15^ Furthermore, this approach provides information on the mechanical stimulation of urethral tissues as a function of device operating conditions. It follows the identification of potential degenerative phenomena.^16^, ^17^ Another advantage of in silico methods is the ability to replicate studies on the same urethra and device, which is not possible experimentally due to biological sample degradation and/or device damage. In this sense, the computational method makes it possible, on the one hand, to replicate and extend the study of a system that is always the same and, on the other hand, to analyse the behaviour of the system in relation to a very wide range of system configurations and loading situations.

One of the main classifications of AUS is extraluminal and intraluminal sphincters.^18^, ^19^, ^25^ The formers are based on hydraulic or magnetic devices that wrap around the urethra and their implantation requires invasive surgery; the sphincter effect of the device involves squeezing of the urethra, lumen occlusion and associated tissue compression.^20^ Intraluminal sphincters are mostly cylindrical devices, usually placed near the bladder neck. The cylindrical body occupies the entire lumen. Urine flow can only develop in the inner region of the cylindrical body when a specific signal, which can be magnetic or electrical, triggers micturition. Implantation of the AUS requires a minimally invasive, non‐surgical procedure. Fixation of the device is usually achieved by means of a proximal shaft structure, which is located inside the bladder, and a distal shaft structure, which fixes the device to the urethral wall.^21^ Particularly with regard to the distal shaft structure, fixation may induce relevant mechanical stimulation of the tissue.

In particular, the following extraluminal and intraluminal sphincter devices are referred to AMS 800 (Boston Scientific Corporation, Marlborough, MA, USA) and Relief (Relief Srl, Pisa, Italy). To date, the functionality and reliability of these devices have mostly been studied using the classical clinical approach, according to the standard regulations for prosthetic devices. On the other hand, this work aims to demonstrate the capabilities of computational methods for the biomechanical analysis of the urethral tissue stimulation due to the AUS action, allowing a comparison between the two devices. A model of the lower urinary tract was provided, and models of both AUS systems were developed. Specific computational analysis strategies were defined to investigate the interaction of the AUS with urethral tissues. The results highlighted the stress and strain fields within the biological tissues and the potential degenerative phenomena that may occur.

## 2 MATERIALS AND METHODS

Computational models of both the urethra and the AUS were developed with the aim of computationally analysing the mechanical effects of the AUS on urethral tissues and structures. The models were then coupled, and specific simulation strategies were adopted.

### 2.1 Urethra model

**FIGURE 1:**
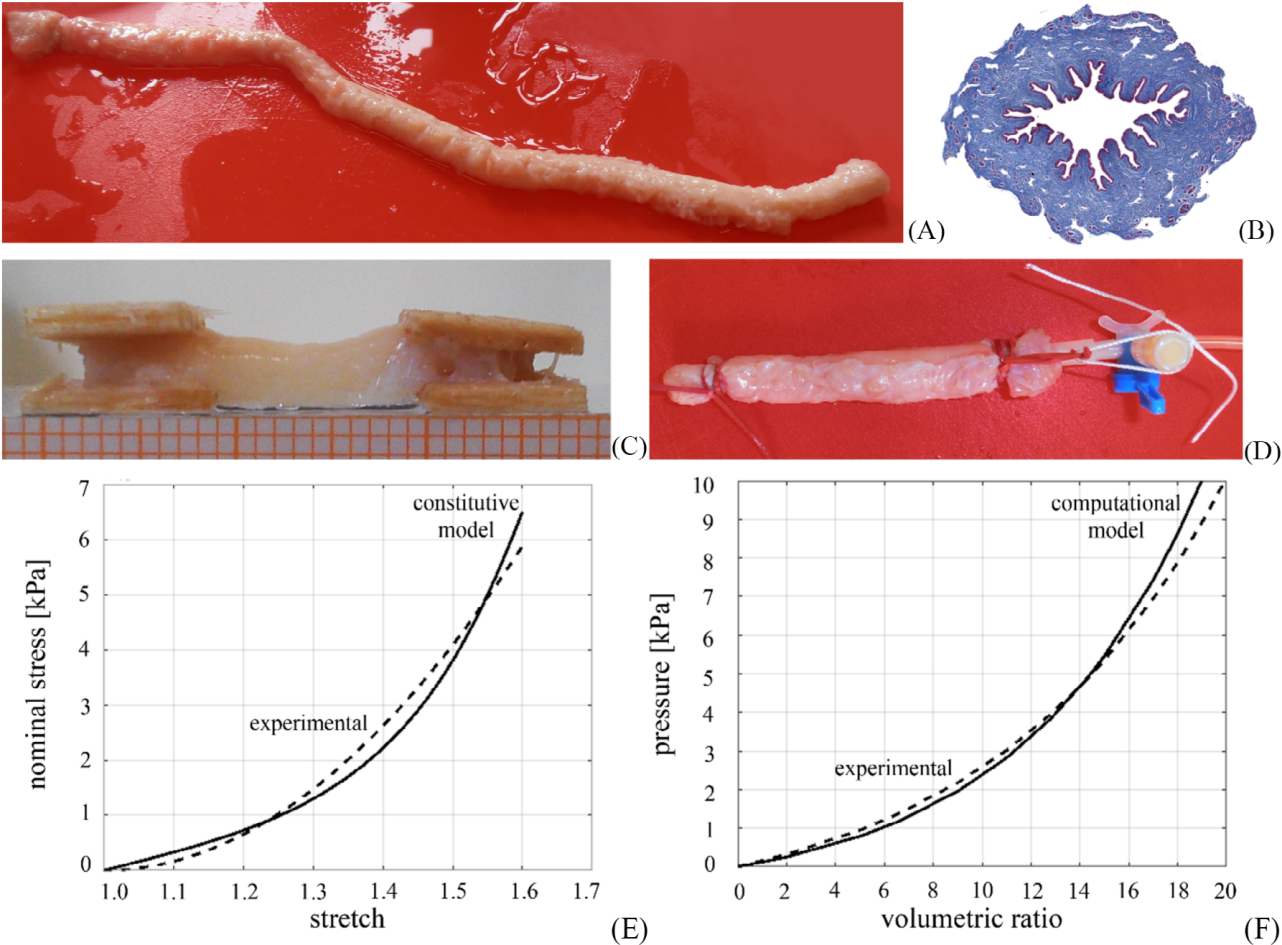
Mechanical characterisation of the urethra: whole urethra sample after dissection (A); typical trichrome histological section showing the urethra conformation: the lumen is surrounded by a thin layer of dense connective tissue and a thick spongiosum stratum (B); experimental samples for tensile tests at the tissue level (C); experimental sample for insufflation tests at the structure level (D); median experimental data from tensile tests (dashed curve) and comparison with constitutive model results (continuous curve) (E); median experimental results from insufflation tests (dashed curve) and comparison with computational model results (continuous curve) (F).

A model of the male urethra was realised based on extensive experimental activities.^22^, ^23^ Due to the difficulty in obtaining sufficiently large and non‐degenerated samples of the human urethra, the equine model was adopted because of its histological and mechanical similarity to human structures. Samples were obtained from a local abattoir and dissected to isolate only the urethra (Figure 1A).

Histological examination revealed a thin layer of dense connective tissue lining the lumen, followed by a thick layer of trabecular meshwork with loose connective tissue and blood vessels (Figure 1B). Mechanical tests were carried out at both tissue and structural levels. The former consisted of tensile and stress relaxation tests (Figure 1C), while the latter consisted of inflation tests on tubular specimens (Figure 1dD). The experiments highlighted the typical features of urethral tissue mechanics, in particular large strain phenomena and non‐linear mechanical response. Processing of tensile and stress relaxation test data resulted in near‐equilibrium stress–strain curves whose inverse analysis allowed the identification of Ogden hyperelastic parameters (Figure 1E). Specifically, the Ogden parameter *μ* was 20 kPa for the dense connective tissue layer and 1.1 kPa for the spongiosum stratum, while the Ogden parameter *α* was 6.178 for both layers. The geometric analysis of the histological sections allowed the development of a finite element model of the urethra, which was used to simulate inflation tests. Comparison between the model and experimental results ensured the reliability of the computational model (Figure 1F).

### 2.2 AMS 800 model interacting with urethra model

**FIGURE 2:**
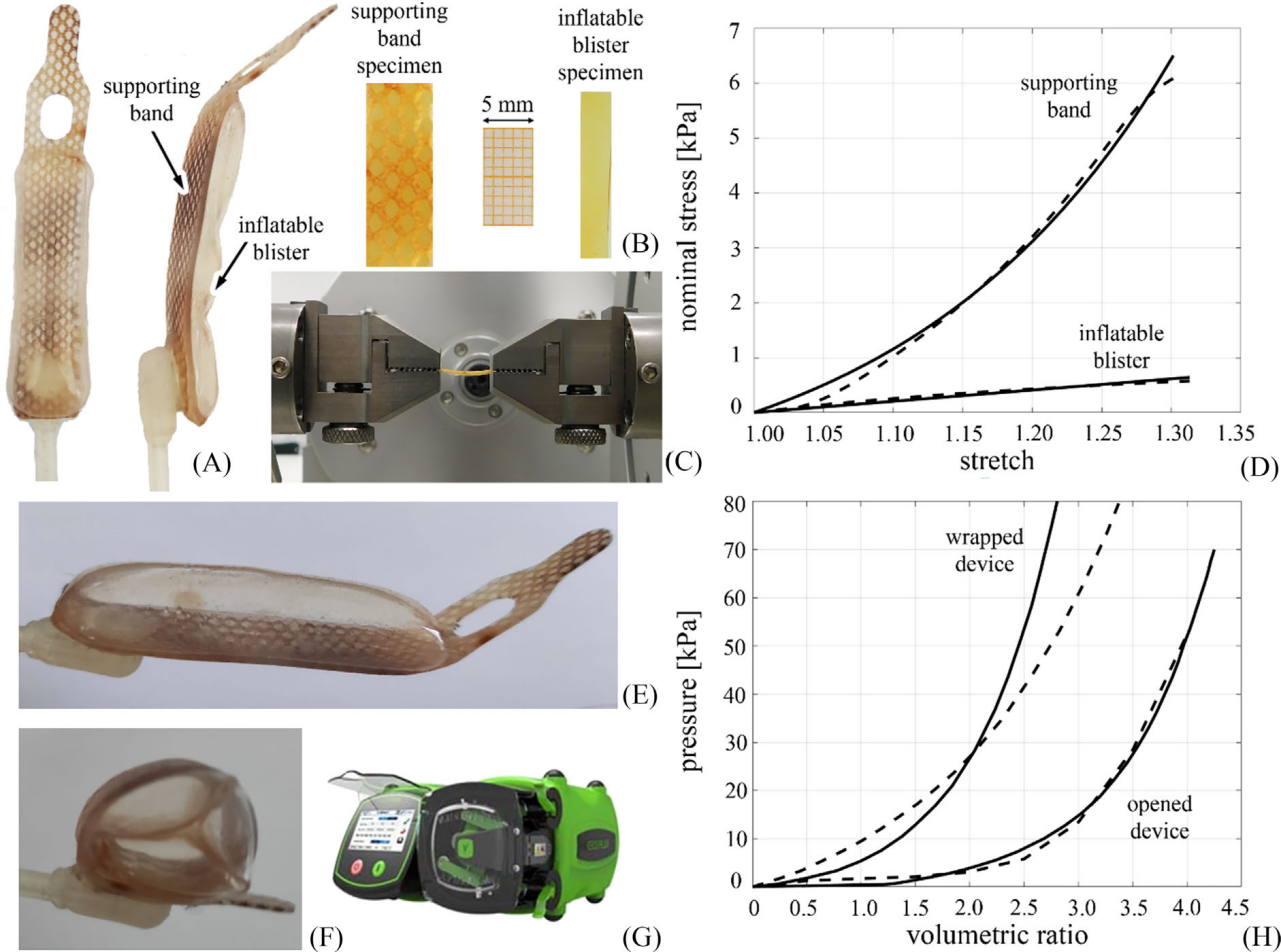
Mechanical characterisation of AMS 800: overview of the device and the components, such as supporting band and inflatable blister (A); experimental specimens for uni‐axial tensile testing of supporting band and inflatable blister materials (B); experimental setup for performing uni‐axial tensile tests (C); experimental results from tensile tests (dashed curves) and data from constitutive identification (continuous curves) for supporting band and inflatable blister materials (D); experimental inflation of AMS 800 in the opened (E) and in the wrapped (F) configurations; peristaltic pump adopted for insufflation tests (G); comparison of experimental (dashed curves) and computational (continuous curves) results from inflation tests on both opened and wrapped configurations of the device (H).

A similar coupled experimental and computational approach was used to define, identify and validate the computational model of the AMS 800.^27^, ^28^ The sphincter device consists of an inflatable rubber blister attached to a fibre‐reinforced supporting band (Figure 2A). The blister is connected to a pump by a flexible tube. After wrapping the device around the bulbar urethra, the blister is inflated with saline solution to a pressure of 6–8 kPa. The urethra is then occluded, and urinary continence is restored. Micturition occurs when the blister is deflated. CT scanning provided images of the device, whose segmentation and CAD processing resulted in a virtual 3D solid model. Mechanical tests at the material level were carried out on samples of both the blister and the supporting band (Figure 2B,C). In particular, constant strain rate tensile tests provided the necessary data to identify hyperelastic parameters (Figure 2D). A neo‐Hookean model characterised the rubber of the blister, while an exponential fibre‐reinforced strain energy function was assumed for the supporting band. Full details of the constitutive analysis of the AMS 800 materials are reported in.^27^ Further experiments were performed at the structural level, such as insufflation tests developed on both linear and wrapped conformations of the device (Figure 2E–G). The computational simulation of the insufflation tests and the comparison with experimental data confirmed the reliability of the model (Figure 2H).

**FIGURE 3:**
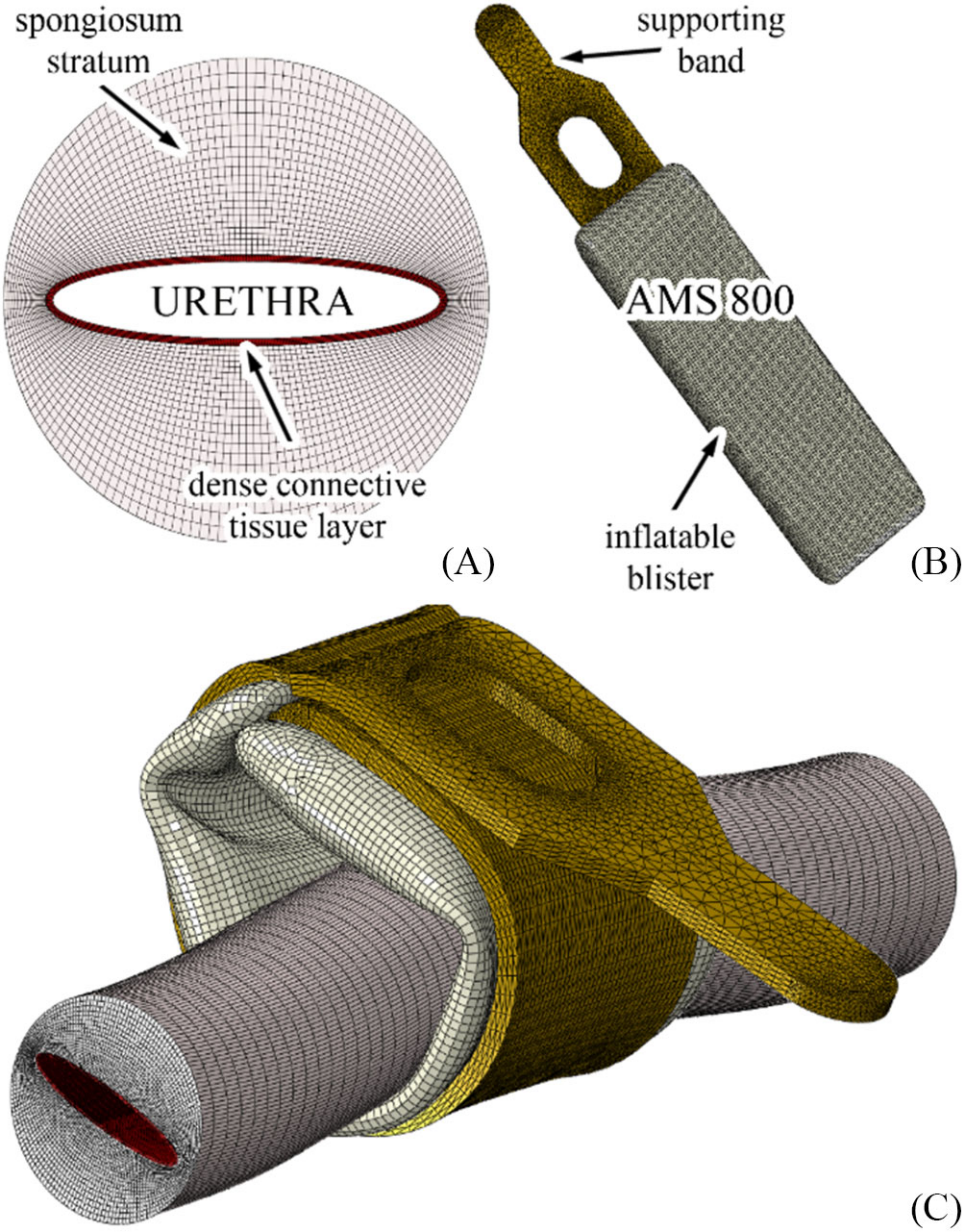
Finite element models of urethra (A), AMS 800 (B) and the coupling of urethra with the wrapped artificial urinary sphincter (C).

Typical urethra and lumen dimensions were assumed to provide a general investigation of the AMS 800 urethra interaction. The urethra model consisted of a 50‐mm‐long, 5‐mm radius cylinder with an elliptical lumen (1.6 and 8 mm minimum and maximum axes respectively) and was finite element modelled using hexahedral elements (Figure 3A). A 0.14‐mm thick layer of dense connective tissue was defined around the elliptical lumen, while a thick layer of spongiosum tissues defined the circular shape. The mechanical behaviour of the dense connective tissue layer and the spongiosum stratum was defined according to the previously identified Ogden formulation. The AMS 800 model (Figure 3B) was positioned to correspond to the central region of the urethra. A contact strategy specified the mechanical interactions between the model surfaces according to a hard contact formulation in the normal direction and a penalty friction formulation in the tangential plane. Specifically, a coefficient of friction of 0.02 was assumed for the self‐interaction of the urethral lumen, while a coefficient of friction of 0.1 characterised the interaction between the other surfaces^8^, ^11^. The outer sections of the urethra were completely fixed to simulate continuity with the other urethral regions. The computational analysis of the interaction between the AMS 800 and the urethra consisted of two steps. The first step considered the AMS 800 wrapping around the urethra (Figure 3C) by means of a specifically defined displacement field, while in the second step, the cuff was progressively insufflated up to a pressure of 8 kPa, defined according to clinical practice.^8^ The strong nonlinearity of the problem, with respect to large displacement and strain phenomena, material nonlinearity, contact and self‐contact conditions, suggested developing the computational simulations in the framework of an explicit solver, such as Abaqus/Explicit 2022 (Dassault Systemes, Vélizy‐Villacoublay, France).

In terms of model complexity, the urethra consisted of 346 569 linear hexahedral elements, corresponding to 397 440 nodes, while the AMS 800 model consisted of 373 911 linear tetrahedral elements for the supporting band and 51 503 linear triangular shell elements (0.4 mm thick) for the blister, resulting in 79 182 and 51 503 nodes respectively. The calculations were performed on an HPC server equipped with four Intel Xeon E7‐8890 v4, 512 GB RAM and SSD HD, using 50 threads, resulting in an average analysis time of 72 h.

### 2.3 Relief model interacting with urethra model

**FIGURE 4:**
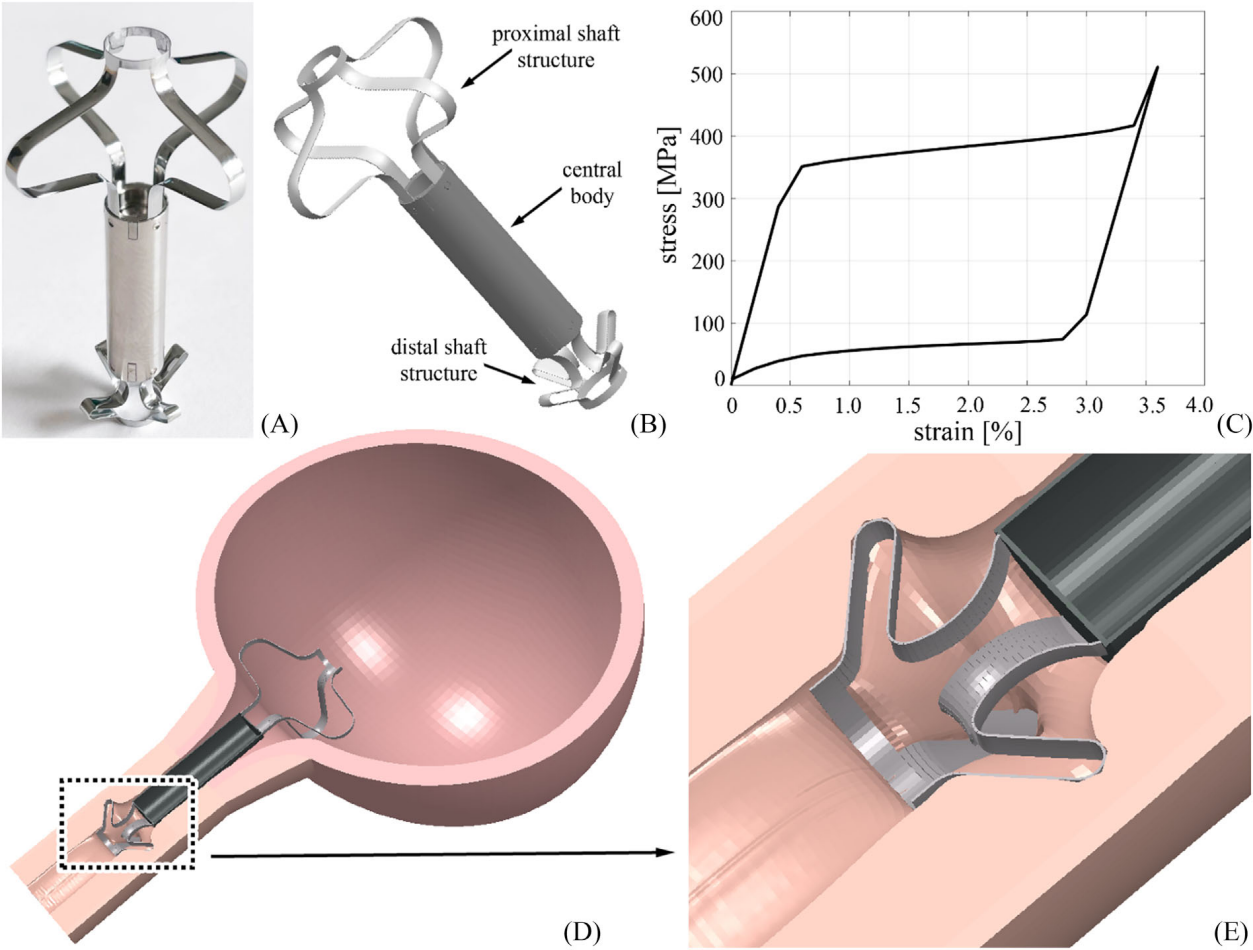
Computational modelling of Relief. Picture of the AUS (A) and virtual solid model (B). Mechanical behaviours of the shape memory alloy that make up proximal and distal shafts (C). Overview of the Relief placement within the lower urinary tract (D) and detail of the region of interaction between the distal shaft and the urethra (E). AUS, artificial urinary sphincters.

Relief is an intraluminal AUS (Figure 4A), which is positioned in the urethra in a minimally invasive manner using standard urological tools such as a cannula for cystoscopy.^21^ The device has an external diameter of 7 mm and is 52.5 mm long. The system is grounded to a magnetic trigger mechanism that allows micturition. More specifically, the device consists of a unidirectional polymeric valve and a safety system that responds to magnetic fields and can control the pressure opening of the valve. The safety system is based on a safety cursor, an internal magnet and a spring. In the absence of external triggers, the safety system remains in the ‘on’ state: The spring preload pushes the safety cursor and forces it into contact with the valve, stiffening it. The valve is thus closed up to a threshold pressure (16 kPa), and continence is maintained. When an external magnet is positioned close to the skin, the internal magnet is attracted towards the skin, in the opposite direction to the valve. As a result, it compresses the spring and causes the safety cursor to move out of contact with the valve. In this way, the valve opens and urination takes place with limited resistance.

The Relief AUS is pinned to the lower urinary tract by two shaft structures (Figure 4B) made of shape memory alloy (SMA) (Figure 4C). These structures consist of four shaft elements and are located at opposite ends of the device. They are initially encapsulated and expand after AUS positioning. Specifically, the proximal shaft structure is located within the bladder to prevent movement of the AUS from the bladder to the urethra. The distal shaft structure is located within the urethra and prevents movement of the device in both directions by stretching the urethral wall (Figure 4D). In terms of mechanical stimulation of the urethral tissues, the area of interaction between the urethra and the distal shaft structure is the most critical (Figure 4E). In particular, a large expansion effect of the shaft structure results in high friction between urethra and shaft and ensures good positioning of the device, but also a relevant mechanical stimulation of the urethral tissues.

With the aim of analysing the interaction phenomena occurring between the Relief device and the urethral tissues, the structural configuration of the AUS device was simplified by considering a linear elastic central cylindrical body (Ti6Al4V with *E* = 114 000 MPa and *ν* = 0.315) and the two SMA shaft structures. The behaviour of the SMA was defined using a specially developed routine, as described in detail in.^24^

AUS is often used after prostatectomy, where the bulbar urethra is connected to the bladder neck. The biological structure interacting with the relief device was therefore composed of the bladder and the bulbar urethra. The mechanical behaviour of the urethral tissue was the same as previously reported, while the mechanics of the bladder tissue was characterised by the hyperelastic formulation and parameters reported by.^26^ Mechanical interactions between AUS and urethral tissues were specified by a hard contact condition along the normal direction, while a smooth contact strategy specified the tangential response. Taking advantage of the symmetric configurations of the biological structure, sphincter device and AUS action, a quarter of the overall model was adopted by imposing specific constraint conditions. Specifically, symmetry constraints avoided rigid body motion along directions perpendicular to the urethral axis, while zero displacement along the urethral axis was imposed on the extreme urethral section. Three main steps defined the analysis of the interaction between the relief device and the bladder‐urethra structure. In the first step, the shaft structures were enveloped and the relief device was placed inside a rigid tube. In the second step, the rigid tube containing the relief device was inserted into the biological structures. Finally, in the last step, the rigid tube was removed, the shaft structures were opened and the Relief device was fixed.

In terms of model complexity, the computational model of the Relief device accounted for 2421 four‐node linear shell elements (0.2 mm thick) for the cylindrical body, while 1840 four‐node linear shell elements defined the shaft structures (0.1 mm thick). In total, 2557 and 2310 nodes characterised the cylindrical body and shaft structures respectively. On the other hand, the bladder–urethra model consisted of 163 900 linear hexahedral elements and 177 143 nodes. The calculations were performed using a 528‐node HPC and AbaqusExplicit 2022 software. Each node was equipped with two Intel Xeon Cascade Lake 8260 with 24 cores each, 384 GB RAM and SSD HD, resulting in a 25‐core allocation and an average analysis time of 20 h.

## 3 RESULTS

**FIGURE 5:**
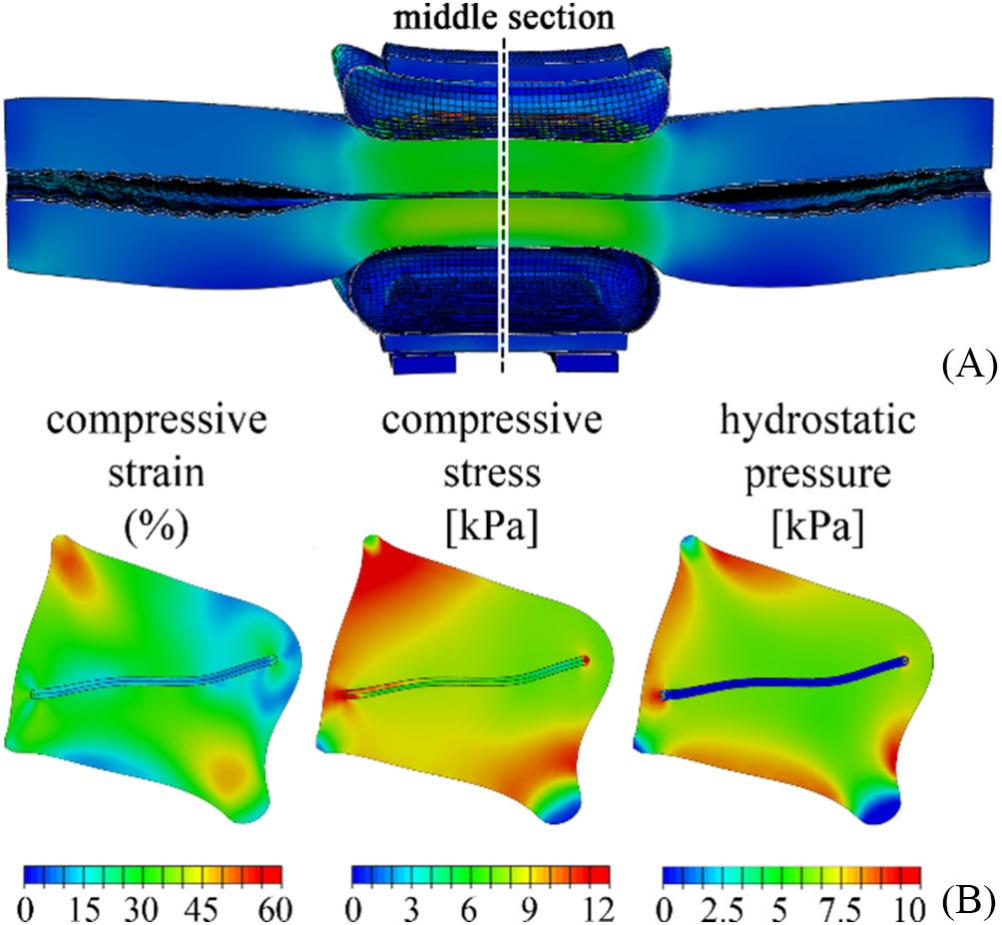
Results from the numerical simulation of urethra occlusion by AMS 800, according to an 8‐kPa cuff pressure. General overview of a longitudinal section showing the occluded region (A). Contours of compressive strain, compressive stress and hydrostatic pressure in the urethra mid‐section (B).

The models developed allowed the functionality of the AUS‐urethra system to be investigated, depending on the specific device, such as the extraluminal AMS 800 and the intraluminal Relief. The finite element studies provided information and data to quantitatively describe the mechanical stimulation of the urethral tissues. With regard to the AMS 800, the most compressed region appeared to be the urethra section in the centre of the AUS. A general view of the occluded region is shown in Figure 5A, while Figure 5B gives an overview of the compressive strain, compressive stress and hydrostatic pressure in the mid‐section.

**FIGURE 6:**
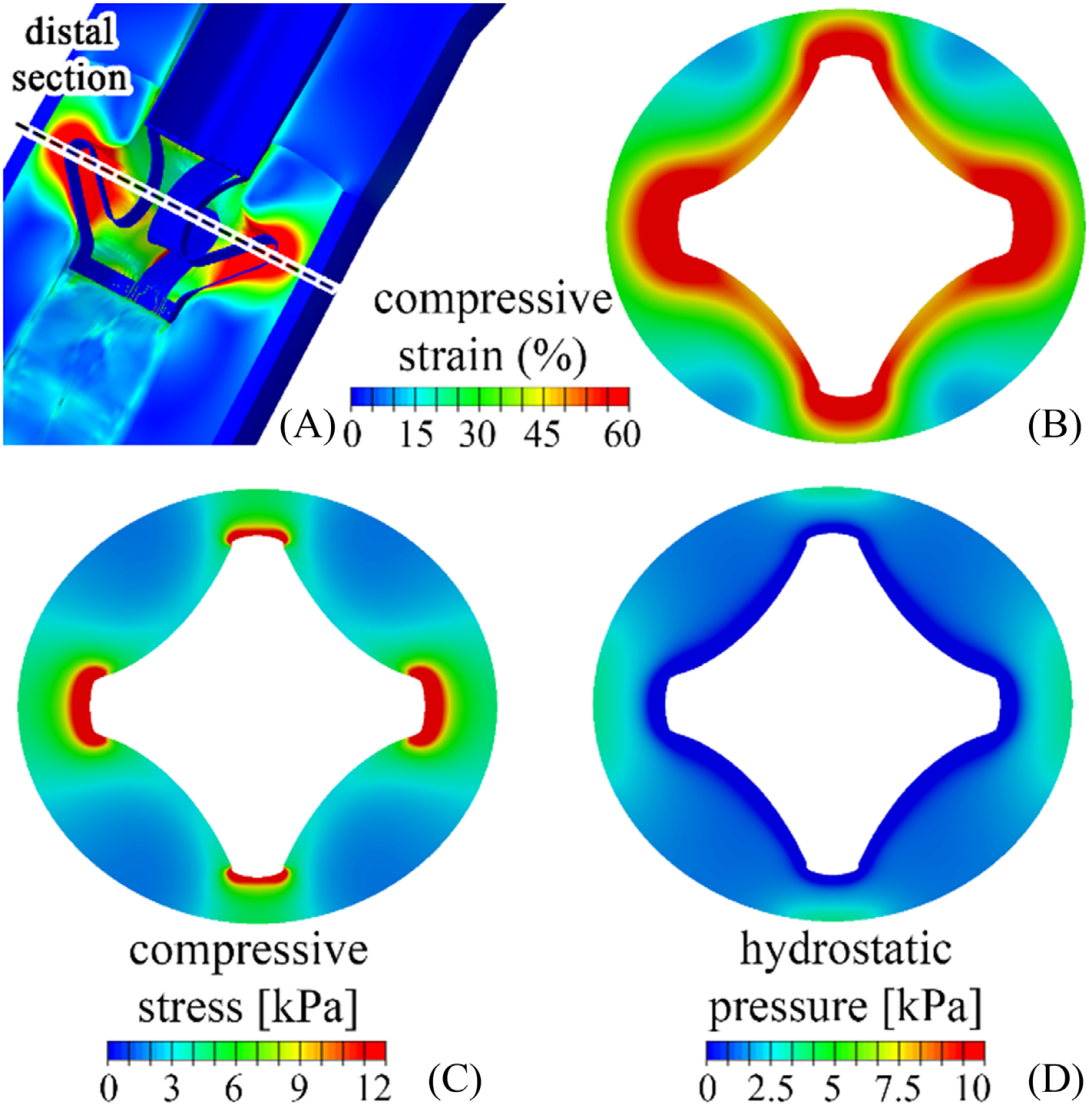
Mechanical stimulation of urethral tissues interacting with the distal shaft structure (A). Details of compressive strain (B), compressive stress (C) and hydrostatic pressure (D) distributions over the most mechanically stimulated section.

In the case of Relief, the greatest compressive stimulation occurred in the positioning region of the distal stem structure. Figure 6 provides an overview of the mechanical quantities in this region (Figure 6A) with details of the distributions of compressive strain (Figure 6B), compressive stress (Figure 6C) and hydrostatic pressure (Figure 6D) over the most mechanically stimulated section.

**FIGURE 7:**
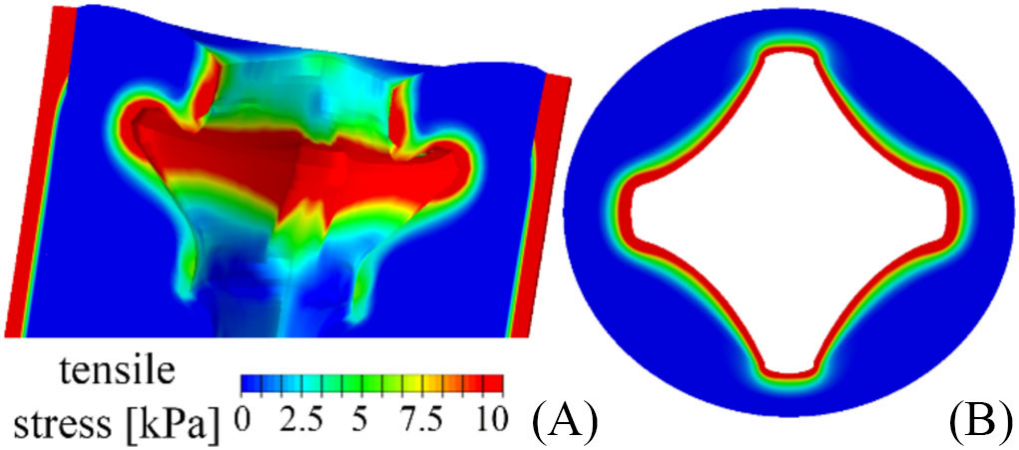
Tensile stimulation of urethral tissues. Distribution of tensile stress over the bladder‐urethra system (A) and detail at the distal shaft structure section (B).

While the AMS 800 is primarily compressive to the urethral tissue, the specific pinning technology of the Relief also provides tensile stimulation. Figure 7 provides an overview of the tensile stress distribution.

**FIGURE 8:**
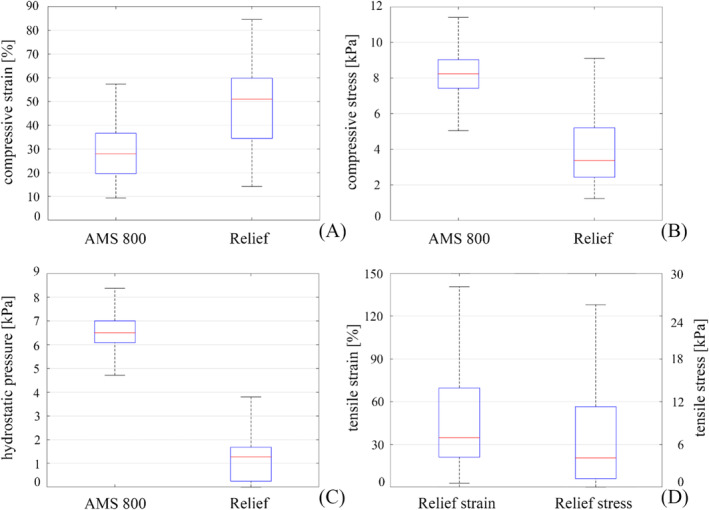
Box plot representation of compressive strain (A), compressive stress (B), hydrostatic pressure (C) and tensile strain and stress (D) for both AMS 800 and Relief devices. Result distributions are reported at the urethra middle section for AMS 800 and at the distal shaft section for Relief. Tensile strain and stress distributions are reported for Relief only, because in the case of AMS 800, the magnitude of tensile strain and stress is almost negligible.

Finally, for a quantitative description of the mechanical parameters, box plots of compressive strain, compressive stress, hydrostatic pressure and tensile stress are shown in Figure 8. The results relate to the mid‐section for AMS 800 and the distal shaft section for Relief. In the case of AMS 800, tensile strains and stresses are almost negligible and the corresponding box plots are not shown.

## 4 DISCUSSION AND CONCLUSION

The activities developed provided an in silico approach to the reliability assessment of two different AUS devices, which can be extended to other types of artificial sphincters. The operating principles of the analysed sphincters, AMS 800 and Relief, are completely different, and therefore, a comparison of performance does not seem possible. However, from the point of view of the reliability of the device, that is its ability not to cause degenerative processes, the computational method allows such a comparison to be made by providing an estimate of the mechanical stimulation of biological tissues.

Specifically, the proposed methodology allowed a quantitative assessment of the mechanical stimulation of the tissues due to the interaction between the AUS and the urethra, providing data to estimate the probability of degenerative effects such as vasoconstriction, inflammation, atrophy and/or erosion. Such phenomena may determine the failure of the device and consequently its removal.^6^

The investigation of AMS 800 and Relief reliability by the in silico approach highlighted the most critical regions of the two systems: the central part of the AUS‐urethra interaction region for AMS 800 (Figure 5) and the distal shaft structure for Relief (Figure 6). With respect to such regions, compressive strains greater than 27.96% (median) were calculated for AMS 800, while compressive stress and hydrostatic pressure were locally greater than 8.23 and 6.50 kPa (median) respectively (Figures 5 and 8). On the other hand, compressive strain greater than 50.96% (median) also characterised the Relief, while compressive stress and hydrostatic pressure were greater than 3.37 and 1.28 kPa (median) respectively (Figures 6 and 8). The lower compressive stress and hydrostatic pressure that characterise the Relief device suggest a reduced risk of vasoconstriction. On the other hand, the pinning mechanism of Relief resulted in tensile strains and stresses within the urethral tissues (Figures 7 and 8) which were greater than 34.72% and 4.1 kPa (median values) respectively. In the case of AMS 800, tensile strains and stresses were almost negligible.

The results highlighted the limitations of the standard design approach to AUS: traditional engineering design (i.e. mechanical or mechatronic) of device functionality, experiments on human cadavers or animal models and finally clinical trials. The main limitations of this approach are the high economic, time and ethical costs; the limited number of situations that can be studied, such as AUS configurations and urethral conformations; and the limited information on mechanical stimulation of urethral tissues.

On the other hand, the in silico study of the interaction between the AUS and the urethra makes it possible to analyse the functionality of the AUS by considering the configuration of the AUS and the conformation of the urethra as parameters. To this end, physics‐based strategies and artificial intelligence techniques can be used to find the optimal solution, taking into account the needs of the individual patient.

In conclusion, the optimal design of an artificial sphincter must necessarily take into account aspects inherent to both the traditional approach, such as in vitro and in vivo experimentation, and advanced engineering methods that allow in‐depth study of the mechanical consequences of sphincter action on urethral tissue functionality.

## AUTHOR CONTRIBUTIONS

Gianluca Mazzucco is the first author and provided for methods definition and results discussion. Emanuele Luigi Carniel provided for ideation and general coordination of the research. Other authors contributed equally to this manuscript.

## CONFLICT OF INTEREST STATEMENT

The authors certify that, although there is a conflict of interest for some authors (Leonardo Marziale, Tommaso Mazzocchi, Gioia Lucarini) because they are members of the company supporting the study (Relief Srl), all analyses, results and conclusions have been fairly and thoroughly reported and were in no way influenced by this conflict of interest. The other authors disclose any financial and personal relationships with people or organisations that could inappropriately influence (bias) their work.
